# Biocontrol by *Fusarium oxysporum* Using Endophyte-Mediated Resistance

**DOI:** 10.3389/fpls.2020.00037

**Published:** 2020-02-06

**Authors:** Francisco J. de Lamo, Frank L. W. Takken

**Affiliations:** Molecular Plant Pathology, Faculty of Science, Swammerdam Institute for Life Sciences, University of Amsterdam, Amsterdam, Netherlands

**Keywords:** *Fusarium*, endophyte, induced resistance, biocontrol, PR protein

## Abstract

Interactions between plants and the root-colonizing fungus *Fusarium oxysporum* (Fo) can be neutral, beneficial, or detrimental for the host. Fo is infamous for its ability to cause wilt, root-, and foot-rot in many plant species, including many agronomically important crops. However, Fo also has another face; as a root endophyte, it can reduce disease caused by vascular pathogens such as *Verticillium dahliae* and pathogenic Fo strains. Fo also confers protection to root pathogens like *Pythium ultimum*, but typically not to pathogens attacking above-ground tissues such as *Botrytis cinerea* or *Phytophthora capsici*. Endophytes confer biocontrol either directly by interacting with pathogens *via* mycoparasitism, antibiosis, or by competition for nutrients or root niches, or indirectly by inducing resistance mechanisms in the host. Fo endophytes such as Fo47 and CS-20 differ from Fo pathogens in their effector gene content, host colonization mechanism, location in the plant, and induced host-responses. Whereas endophytic strains trigger localized cell death in the root cortex, and transiently induce immune signaling and papilla formation, these responses are largely suppressed by pathogenic Fo strains. The ability of pathogenic strains to compromise immune signaling and cell death is likely attributable to their host-specific effector repertoire. The lower number of effector genes in endophytes as compared to pathogens provides a means to distinguish them from each other. Co-inoculation of a biocontrol-conferring Fo and a pathogenic Fo strain on tomato reduces disease, and although the pathogen still colonizes the xylem vessels this has surprisingly little effect on the xylem sap proteome composition. In this tripartite interaction the accumulation of just two PR proteins, NP24 (a PR-5) and a β-glucanase, was affected. The Fo-induced resistance response in tomato appears to be distinct from induced systemic resistance (ISR) or systemic acquired resistance (SAR), as the phytohormones jasmonate, ethylene, and salicylic acid are not required. In this review, we summarize our molecular understanding of Fo-induced resistance in a model and identify caveats in our knowledge.

## Introduction

The *Fusarium oxysporum* species complex embraces a variety of strains ubiquitously present in soils. Most of these strains are saprotrophs and despite their ability to colonize plant roots the majority represents commensal endophytes not affecting plant fitness (Bao et al., 2004). Some *F. oxysporum* (Fo) strains, such as Fo47 and CS-20, are actually beneficial to the host and can provide protection against root pathogens (**Table 1**). Biocontrol-conferring Fo strains, such as Fo47, have been identified in vascular wilt-disease suppressive soils (Alabouvette, 1986). Identification of the causal microbes in wilt suppressive soils is typically done by sterilizing the soil following subsequent re-inoculation with the original microbes and screening for isolates that restore the suppressive effect against *Fusarium* wilt (Tamietti et al., 1993).

**Table 1:** *Fusarium oxysporum* (Fo)-mediated biocontrol in various plant species.

Fo	Host plant	Pathogen (P)	Inoculation method	Protected organ	Biocontrol mechanism	Publication
Fon (60-3A) Fon (ATCC 18467) Foc (ATCC 16416)	Watermelon (*C. lanatus*)	*Fon* ATCC 62940 *C. lagenarium*	Fo: root inoc *Fo* f.sp. *niveum*: root inoc *C. lagenarium*: leaf inoc	Root and shoot	Induced resistance	(Biles and Martyn, 1989)
Fo f.sp. *dianthi* (WCS816)	Tomato (*S. lycopersicum*)	Fol (WCS 801)	Co-inoc +split root system	Root	Induced resistance	(Kroon et al., 1991)
*5a1* *T* *Fop2* *11V* *N1.5*	Tomato (*S. lycopersicum*)	Fol	Fo: soil inoc P: root inoc	Root	Induced resistance	(Tamietti et al., 1993)
Fo47	Tomato (*S. lycopersicum*)	Fol8 and Fol8B	Soil inoc ± split root system	Root	Induced resistance	(Fuchs et al., 1997)
Fo47 CWB 306 CWB 307 *	Asparagus (*A. officinalis*)	Foa	Soil inoc	Root	Competition for nutrients	(Blok et al., 1997)
Fol218 Fon23M Fon18M	Tomato (*S. lycopersicum*) Muskmelon (*C. melo*)	Fol4287 Fon23M Fon18M Fon11-27	Fo: root (co)inoc P: root inoc	Root	Induced resistance and competition	(Huertas-Gonzalez et al., 1998)
Fo47	Tomato (*S. lycopersicum*)	Fol32	Fo: soil pre-inoc ± split root system P: soil inoc	Root	Induced resistance and antagonism	(Duijff et al., 1998)
Fo47	Tomato (*S. lycopersicum*)	Fol8	Soil pre-inoc	Root	Induced resistance	(Fuchs et al., 1999)
Fo47 **	Flax (*L. usitatissimum*)	Foln3GUS	Co-inoc	Root	Competition for carbon and iron	(Duijff et al., 1999)
Fo47 CS-20	Tomato (*S. lycopersicum*) Watermelon *(C. lanatus)*	Fol IA-7 Fob MD-1 Fon CS93-8	Fo: soil pre-inoc ± split root system P: soil inoc	Root	Induced resistance and competition	(Larkin and Fravel, 1999)
Fo47	Eucalyptus (*E. viminalis*)	Foeu1	Root co-inoc	Root	Competition for infection sites	(Salerno et al., 2000)
CS-20	Basil (*O. basilicum*)	Fo-B1	Fo: Drench inoc P: present in seeds	Root	Induced resistance	(Fravel and Larkin, 2002)
CWB312 CWB314 CWB318	Asparagus (*A. officinalis*)	Foa	Fo: soil pre-inoc ± split root system P: soil inoc	Root	Induced resistance	(He et al., 2002)
Fo47	Cucumber (*C. sativus*)	*P. ultimum* (BARR 447)	Fo: soil pre-inoc P: root inoc	Root	Antibiosis, mycoparasitism and induced resistance	(Benhamou et al., 2002)
Fo47	Flax (*L. usitatissimum*)	Foln3	Soil co-inoc	Root	-	(Trouvelot et al., 2002)
CS-20 CWB312 CWB314 CWB318 Fo47	Asparagus (*A. officinalis*)	Foa	Fo: root/soil pre-inoc P: soil inoc	Root	Induced resistance and competition	(Elmer, 2004)
205 Fo from tomato fields in Florida *	Tomato *(S. lycopersicum)*	Fol 32SK-3	Fo: soil inoc P: present in seeds	Root	Antagonism	(Bao et al., 2004)
Fol (ATCC 48112)	Pepper (*C. annuum*)	*P. capsici* UDC1PC *V. dahliae* *B. cinerea* B0510	Fo: root pre-inoc *V. dahliae*: root inoc *P. capsici*: soil inoc *B. cinerea*: leaf drop-inoc	Root and Shoot	Induced resistance	(Díaz et al., 2005)
Fo47	Tomato (*S. lycopersicum*)	Forl (ZUM 2407)	Soil co-inoc	Root	Induced resistance and competition	(Bolwerk et al., 2005)
CAV 255 CAV 241 Fo47 *	Banana (*M. acuminata*)	Fo f.sp. *cubense* (CAV 045)	Fo: soil pre-inoc P: soil inoc	Root	–	(Nel et al., 2006)
Fo47	Tomato (*S. lycopersicum*)	Fol8	Soil co-inoc	Root	Competition for nutrients	(Olivain et al., 2006)
Fo52 Fo47 Fo47b10	Chickpea (*C. arietinum*)	Fo f.sp. *ciceri*	Fo: soil pre-inoc ± split root system P: soil inoc	Root	Induced resistance	(Kaur and Singh, 2007)
Fo47	Tomato (*S. lycopersicum*)	Fol8	Root co-inoc	Root	competition	(Nahalkova et al., 2008)
Fo47 ***	Tomato (*S. lycopersicum*)	*P. oligandrum* *B. cinerea*	Root co-inoc	Root and Shoot	Antibiosis, mycoparasitism and induced resistance	(Le Floch et al., 2009)
Fo (F2)	Eggplant (*S. melongena*)	*V. dahliae*	Fo: root pre-inoc ± split root system P: root inoc	Root	competition	(Pantelides et al., 2009)
Fo47	Pepper (*C. annuum*)	*V. dahliae* UDC53Vd *P. caspici* PC450 *B. cinerea* B0510	Fo: root pre-inoc *V. dahliae*: root inoc *P. capsici*: soil/leaf inoc *B. cinerea*: leaf drop-inoc	Root	induced resistance and antagonism/competition	(Veloso and Díaz, 2012)
Fo47	Tomato (*S. lycopersicum*)	Fol8	Fo: root pre-inoc P: root inoc	Root	Induced resistance	(Aimé et al., 2013)
CS-20	Cucumber (*C. sativus*)	Foc	Fo: root pre-inoc P: root inoc	Root	Induced resistance	(Pu et al., 2014)
Fo47	Tomato (*S. lycopersicum*)	Forl12	Fo: soil pre-inoc ± split root system P: soil inoc	Root	Induced resistance and competition	(Aïcha et al., 2014)
Fo47	Pepper (*C. annuum*)	*V. dahliae* (UDC53Vd)	Fo: root pre-inoc P: root-inoc	Root	Induced resistance and competition	(Veloso et al., 2016)
Fo47	Tomato (*S. lycopersicum*)	Fol007	Root co-inoc	Root	Induced resistance and competition	(de Lamo et al., 2018)
Fo47 ****	Watermelon (*C. lanatus*) Cotton (*Gossypium* sp.) Eggplant (*S. melongena)*	*Fon* Fo f.sp. *vasinfectum* *V. dahliae*	Soil inoc	Root	Competition	(Zhang et al., 2018)
Fo47	Tomato (*S. lycopersicum*)	Fol4287	Root co-inoc	Root	Induced resistance and competition	(Constantin et al., 2019)

Fo endophytes are depicted in the first column; root or soil inoculation is abbreviated as root- or soil inoc; when endophytes and pathogen have been inoculated separately they are abbreviated as “Fo” and “P” respectively; otherwise as co-inoc, ± split root system indicate that both setups have been used in the study. Inoculations are typically performed by incubating roots in a spore suspension or by adding spores to the soil as indicated.

*Screening including several Fo strains.

**Fo47 inoculum also tested in combination with Pseudomonas sp.

***Fo47 inoculum mixed with Trichoderma harzianum.

****Fo47 inoculum also tested in combination with different actinomycete bacteria.

Fusarium wilt is one of the major diseases caused by pathogenic Fo strains. Wilts are a major threat for agriculture (Fisher et al., 2012) and Fo ranks among the 10 most devastating fungal plant pathogens worldwide (Dean et al., 2012). Besides wilt disease some strains can also cause foot- or root-rot resulting in serious yield losses in affected crops (Michielse and Rep, 2009). Fo produces micro- and macroconidia and chlamydospores that can remain viable in infected soils for decades, thereby frustrating crop rotation schemes (Nelson, 1981). Pathogenicity of Fo is host-specific, as typically strains infecting one plant species do not cause disease in others. Based on this host-specificity, pathogenic strains have been classified into so-called *formae speciales* (ff.spp.), of which over 100 have currently been described (Armstrong and Armstrong, 1981). An explanation for the emergence of host-specific pathogenic strains may be the extensive use of monocultures with limited crop rotation serving as breeding grounds for pathogens (Xiong et al., 2016). The evolved Fo pathogens can give rise to devastating crop losses, *Fusarium* wilt disease of banana, caused by Fo f.sp. *cubense*, being a prime example (García-Bastidas et al., 2014; Ordoñez et al., 2016).

To control wilt diseases different strategies are currently being employed in agriculture. One of these is chemical control, which includes broad-spectrum biocides like methyl bromide, benomyl, or carbendazim applied before planting. These chemicals can prevent infection, but do not cure a plant once infected. A caveat of these compounds is that they also affect beneficial soil microbiota and some accumulate in the food chain and for this reason many of these products are, or will be, banned (Lopez-Aranda et al., 2016). Heat sterilization of soils overcomes some of these drawbacks, but has the disadvantage that it is non-selective and it can have undesired effects on soil quality (Mahmood et al., 2014). Use of resistant plant varieties, e.g. plants carrying resistance genes is currently the most effective in terms of economy, ecology, and disease control. However, genetically encoded resistance is seldom durable and sooner or later new races emerge that overcome resistance in a never-ending arms race between Fo and its host (Takken and Rep, 2010; de Sain and Rep, 2015). Furthermore, Fo resistance genes are not available in the germplasm of all crops or they cannot be introgressed by breeding (Ploetz, 2015).

The limitations of the current approaches of wilt disease control urges the need to develop alternatives. An interesting alternative strategy is the use of beneficial Fo strains that confer biocontrol and thereby reduce disease incidence. A major advantage of biocontrol is the relatively broad-spectrum- and non-race specific protection conferred by endophytic strains (**Table 1**). A limitation is that the protection provided by these biological agents is highly variable and not consistent between seasons, crops, or fields. As illustration, even in greenhouse trials using tomato plants artificially co-inoculated with a pathogenic and a biocontrol Fo strain significantly different degrees of protection where observed in subsequent years (Fuchs et al., 1999). Furthermore, biocontrol observed under controlled lab conditions is not necessarily scalable to field conditions. For example, controlled soil co-inoculation of asparagus with Fo47 and Fo f.sp. *asparagi* (Foa) resulted in partial disease protection under lab conditions, but application of Fo47 in Foa-infested greenhouses did not reduce wilt disease (Blok et al., 1997).

A better understanding of the molecular mechanisms underlying biocontrol conferred by endophytic Fo strains may help to unleash the full potential that these organisms harbor to control disease conferred by their brothers in crime. In this review we mostly focus on two endophytic Fo strains, Fo47 and CS-20, as these are the best studied strains. We assess the differences between pathogenic and endophytic strains at their root colonization behavior, at the genome level and the responses they trigger in plants. Endophyte-mediated biocontrol consists of two components. The first is based on a direct activity on the pathogenic strain *via* parasitism and antibiosis (Benhamou et al., 2002; Le Floch et al., 2009) or by competing for nutrients or root niches. Several excellent reviews are available describing these non-plant mediated processes (Fravel et al., 2003; Alabouvette et al., 2009; Vos et al., 2014; Latz et al., 2018). In this review we focus on the other component of biocontrol, the indirect plant-mediated resistance response triggered by Fo endophytes, called endophyte-mediated resistance (EMR).

## *F. oxysporum* Confers Biocontrol in Various Plant Species Against Root Pathogens

The ability of a large variety of endophytic Fo strains to confer biocontrol has been reported in many independent studies implying that it is a generic feature for Fo (**Table 1**). This idea is supported by a study in which over 200 different non-pathogenic Fo strains isolated from a tomato field were able to confer biocontrol in tomato, albeit to various degrees (Bao et al., 2004). Another observation is that Fo-based biocontrol is effective in a wide variety of plant species including both monocot and dicot species. This suggests that biocontrol is an ancient property as these families diverged over 200 million years ago (Wolfe et al., 1989). A number of oomycete-caused diseases can also be suppressed by Fo. For instance, Fo47 is reported to reduce disease incidence caused by *Pythium oligandrum* in tomato (Le Floch et al., 2009), *Pythium ultimum* in cucumber (Benhamou et al., 2002) and *Phytophthora capsici* in pepper (Veloso and Díaz, 2012). A common property among these pathogens is that they infect roots, but unlike most pathogenic Fo strains not all colonize the vasculature, implying that EMR is not vasculature-specific.

Two studies report on Fo-induced biocontrol that is not exclusively targeted against a root pathogen (**Table 1**). Pre-inoculation of watermelon roots with *Fo* f.sp. *cucumerinum* (Foc) (a pathogen on cucumber) reduced lesion sizes of *Colletotrichum lagenarium* infected leaves (Biles and Martyn, 1989). The other example details enhanced tolerance to *Botrytis cinerea* in pepper plants pre-inoculated with Fol (a tomato pathogen) (Díaz et al., 2005). Based on our literature survey Fo-induced EMR appears to be mostly root-confined.

## The Root Colonization Pattern of *F. oxysporum* Pathogens Differs From That of Endophytes

Root colonization by Fo endophytes and pathogens has been extensively studied. In this chapter, we compare the root colonization process of Fo endophytes with that of pathogens. In our comparison we include colonization of ‘incompatible interactions’ in which a pathogenic Fo strain colonizes a resistant host, which does not result in disease emergence.

### Spore Germination

The first stage of the colonization process starts with Fo spores or hyphae that grow in the vicinity of a root. Addition of sugar to the soil induces chlamydospore germination of pathogenic Fol and Fo f.sp. *basilici* (Larkin and Fravel, 1999). In natural settings root exudates presumably provide these carbohydrates as exudates from different crops enhance germination of microconidia of Fol and Fo f.sp. *radicis-lycopersici* (Forl) pathogens (Steinkellner et al., 2005). Hyphal exudations may also play a role in conidia germination as Fo uses autocrine pheromone signaling to control germination in a conidial-density dependent manner (Vitale et al., 2019). Some root pathogens effectively grow towards roots using chemotropism (Yao and Allen, 2006). While earlier studies found no evidence of chemotaxis toward tomato roots by Fo47 or the pathogens Fol or Forl (Steinberg et al., 1999; Olivain et al., 2006) a recent study showed that peroxidases secreted by tomato roots elicit Fol chemotropism towards roots (Turrà et al., 2015; Nordzieke et al., 2019). Altogether, it seems that root exudations trigger spore germination and induces directional mycelial growth. At this pre-colonization stage, no differences are noticeable between endophytic and pathogenic Fo strains.

### Host Colonization

Upon germination, both Fo endophytes and pathogens colonize the root surfaces of host and non-host plants. Contact with the root triggers hyphal branching, after which Fo produces hyphal swellings to invade the root. The fungal hyphae enter plant roots *via* wounds, cracks in the epidermis, lateral root emergence points, or by direct penetration of the root tip depending on the Fo strain and plant species involved. Hyphae reach the vascular stele *via* the apoplast of the root cortex. In some cases, intracellular growth is noticeable along with local host cell-death, a phenomenon observed more often among non-pathogenic strains (He et al., 2002; Olivain et al., 2003; Humbert et al., 2015; Gordon, 2017). Both pathogenic and non-pathogenic strains colonize the root cortex (Gordon, 2017), but although the initial colonization pattern is similar, the extent and pattern of colonization differs during later stages. The amount of biomass of a pathogenic strain in the root is typically higher than that of an endophyte. This difference is already apparent at early stages. At 48 h post-inoculation (hpi) higher amounts of fungal biomass for the Fo40 pathogen were detected in roots of soybean plants than for the endophytic Fo36 (Lanubile et al., 2015). A similar difference was reported for other systems, like the interaction between tomato and Fo47 or Forl. Two weeks post-inoculation Fo47 biomass was 10-fold less than that of the pathogen (Validov et al., 2011). These observations imply that in early stages of the interaction Fo endophytes are less efficient root colonizers than pathogens.

Besides the amount of fungal biomass also the root colonization pattern differs between Fo pathogens and endophytes. Typically, only pathogenic strains are able to reach the xylem vessels from where they colonize above-ground tissues. The induced occlusions of the xylem vessels, aimed to restrict pathogen progress, results in the classical wilting symptoms of infected plants (Gordon, 2017). A well-studied example is the interaction between pea roots, Fo47, and the pathogen Fo f.sp. *pisi*. Whereas Fo47 colonization is restricted to the root surface and outermost cell layers of the cortex, the pathogen massively invades the deeper root tissues including the vasculature (Benhamou and Garand, 2001). A similar pattern is seen upon Fo colonization of tomato. When grew in hydroponics, Fo47 and Fol8 both efficiently colonized the surface of tomato tap roots following attachment of the microconidia to the root hair zone. Subsequently, both strains grew towards the elongation zone until they reached the root apex. Whereas Fol8 intensively colonized the deeper root tissues and eventually reached the vasculature, the endophyte was confined to the epidermis and cortex (Nahalkova et al., 2008). In line with these observations, Fo47 proteins were not identified in xylem sap of Fo47-inoculated soil-grown tomato plants, whereas Fol proteins were detected in sap of Fol-infected plants (de Lamo et al., 2018). This difference indicates that even at later stages of root colonization Fo47 does not reach the vasculature. Contrarily, Fo47 was reported to colonize xylem vessels of eucalyptus (Salerno et al., 2000) and the Fo endophyte CS-20 was found to colonize xylem vessels of cucumber (Pu et al., 2014). An explanation for the vascular presence of the latter two endophytic strains could be the clipping of the roots prior to inoculation, providing direct vascular access to the endophyte.

In incompatible interactions, the first stages of the root colonization pattern are similar to that of a compatible interaction. The pathogen colonizes the root cortex, but in contrast to a purely endophytic strain, the pathogen frequently reaches the vasculature of a resistant host. Examples of vascular colonization of a resistant host are chickpea and tomato roots inoculated by either avirulent Fo f.sp. *ciceris* or Fol (Mes et al., 2000; Jimenez-Fernandez et al., 2013; van der Does et al., 2018). Recently vascular colonization of tomato plants carrying three different classes of resistance (*R*) gene types by Fol was compared. Although the plasma membrane-localized immune receptors (I and I-3) restricted colonization to a larger extent than the intracellular receptor (I-2), vascular colonization was observed in all cases (van der Does et al., 2018). The amount of fungal biomass in a resistant plant, however, is low and fungal proteins cannot be detected in the xylem sap of infected plants (de Lamo et al., 2018). These findings are in line with the low number of hyphae observed in vessels of a resistant tomato variety (Mes et al., 2000). Inoculation of resistant cabbage roots with Fo f.sp. *conglutinans* also resulted in marginal colonization of the vasculature and fungal proteins were not identified in the xylem sap either (Pu et al., 2016).

The general pattern is that Fo endophytes, similar to endophytes such as *Serendita indica* (Jacobs et al., 2011) and arbuscular mycorrhizal fungi (Gadkar et al., 2001), are mostly root surface- and cortex-colonizers. Extensive colonization of the root cortex and vasculature is typically restricted to pathogens, a property that correlates with enhanced secretion of cell wall-degrading enzymes by these strains (Jonkers et al., 2009). Pathogenic strains are also able to enter and, to a limited extent, colonize the vasculature of a resistant host. Upon (co-)inoculation of an endophyte and a pathogen both strains coincide at the same root tissues during the early stages of the interaction, but become spatially separated when the pathogen invades the vascular bundle. Therefore, disease protection induced by Fo endophytes at these later stages is likely plant-mediated.

## *F. oxysporum* Endophytism and Pathogenicity Are Genetically Determined by the Fungus–Host Combination

Bioassays can reveal whether a strain is pathogenic on a specific host, or on a specific variety of that host, but these assays cannot establish whether a strain is non-pathogenic. Given the narrow host-range of pathogenic Fo strains (mostly restricted to single plant species) it would be necessary to inoculate a particular strain on all possible plant species and varieties to conclude that it is likely a non-pathogen in case of a negative outcome. Giving the impracticality of such an approach, and the limitation of classical taxonomic features, there has been ample focus on identifying molecular features that could be used to distinguish ff.spp. and to diagnose pathogenic isolates and discriminate them from non-pathogenic strains.

Phylogenetic analyses of the Fo species complex using conserved gene sequences such as those encoding elongation factor 1α typically result in phylogenetic trees in which Fo pathogens and endophytes are distributed together over different clades (Wong and Jeffries, 2006; Ellis et al., 2014; Pinaria et al., 2015). Likewise, trees based on genomic markers such as restriction fragment length polymorphism of the ribosomal intergenic spacer regions, or on mating type results in trees in which the ff. spp. are polyphyletic and cluster together with Fo endophytes in various clades (Alves-Santos et al., 1999; Abo et al., 2005; Ellis et al., 2014; Nirmaladevi et al., 2016). Even a multiple-sequence alignment of 441 conserved core genes from various Fo genomes did not result in a tree that enabled differentiating ff. spp. or allowed unambiguous identification of non-pathogenic strains (van Dam et al., 2018).

A decade ago, however, it was reported that the presence of a lineage-specific chromosome determines pathogenicity of Fol toward tomato (Ma et al., 2010). Horizontal transfer of a pathogenicity chromosome from Fol to Fo47 turned the endophyte into a tomato pathogen (Ma et al., 2010). Subsequent studies revealed that chromosome transfer from a cucurbit-infecting Fo strain could transform Fo47 into a cucurbit pathogen (van Dam et al., 2017). *Vice versa*, loss of a dispensable pathogenicity chromosome from a Fol strain resulted in loss of pathogenicity (Vlaardingerbroek et al., 2016). Hence, pathogenicity appears to correlate with the presence of a pathogenicity chromosome. These pathogenicity chromosomes differ from core chromosomes by a high content of transposable elements and a low gene density (Ma et al., 2010). Genetic analysis of Fol revealed that its pathogenicity chromosome carries the genes encoding the putative host-specific virulence proteins (effectors) that the fungus secretes in the tomato xylem sap following infection (Schmidt et al., 2013). Some of these Secreted In Xylem, or SIX, proteins such as SIX1 (Avr3) (Rep et al., 2004), SIX3 (Avr2) (Di et al., 2017b), SIX4 (Avr1) (Houterman et al., 2008) and SIX6 (Gawehns et al., 2014) are genuine effectors and contribute to fungal virulence on tomato. Many of these effectors were found to be specific for the tomato-infecting strain, providing the means to identify this pathogen based on its effector profile. Based on the features of these Fol *SIX* effector genes an effector prediction pipeline could be constructed in which putative effector genes in Fo can be identified based on: 1) a relatively small size (>25 aa and <300 bp), 2) presence of a signal peptide for secretion, and 3) proximity to a “miniature impala” transposable element (van Dam et al., 2016). Analyses of predicted Fo effectoromes revealed that these are shared between strains from a f.sp. infecting the same host, while they are divergent for those infecting other plant species, thereby allowing distinction of ff.spp. based on their effector profiles (Lievens et al., 2009; van Dam et al., 2016; van Dam et al., 2018). Hence, in contrast to phylogenetic analyses, Fo effectorome exploration proves a powerful tool to predict pathogenicity and the potential plant host for a given strain. Whether a potential pathogen is indeed able to cause disease ultimately depends on the corresponding genotype of the host. If the host carries a resistance gene recognizing a specific effector of the pathogen this may result in activation of gene-for-gene-based resistance response restricting host colonization (Flor, 1956; Jones and Dangl, 2006). For instance, the Fol effector proteins Avr1, Avr2, or Avr3 are recognized by the tomato resistance proteins I, I-2, or I-3 (Simons et al., 1998; Catanzariti et al., 2015; Catanzariti et al., 2017) resulting in the activation of a resistance response in plants carrying these genes (Houterman et al., 2008). Similarly, the effector protein AvrFom2 from the melon pathogen Fo f.sp. *melonis* can be recognized by the melon resistance protein Fom2 thereby conferring avirulence to the fungus (Risser et al., 1976; Schmidt et al., 2016).

Non-pathogenic strains share a set of conserved putative effector genes with pathogenic strains, but typically carry much fewer effector candidates and no or few host-specific effectors (van Dam et al., 2016). This notable difference provides a means to distinguish potential pathogens from non-pathogens by the number of candidate effectors they carry. It is tempting to speculate that the candidate effectorome of non-pathogens determines their capacity to colonize roots and confer EMR. Unfortunately, little is known of the role of effectors for Fo endophytes. One putative effector, CS20EP, of the EMR-conferring CS-20 strain was reported to trigger a defense response in tomato against Fol when applied prior to inoculation with the pathogen (Shcherbakova et al., 2015). The protein was identified in the culture filtrate of *in vitro*-grown fungus. However, whether the *CS20EP* gene is actually expressed during host root colonization awaits future study, as does its role in EMR, for which a knockout strain should be assessed. Altogether, the predicted effector profile from a Fo strain allows its classification as a likely endophyte or as a putative (a)virulent pathogen on a given host. The increasing number of Fo genomes becoming available allows f.sp.-specific effector candidates to be identified and to more precisely predict host-specific pathogenicity of a given strain. Functional analysis of these effectors, and identification of their host targets, could provide new leads to combat pathogens (Gawehns et al., 2013).

## The Timing and Amplitude of Root Responses Upon Colonization by Endophytic or Pathogenic *F. oxysporum* Differ

Plant roots are typically exposed to a highly diverse soil microbiota (Hacquard et al., 2017). Plants recognize microorganisms *via* microbe-associated molecular patterns (MAMPs) that are present in both pathogens and non-pathogens (Henry et al., 2012). Well-known fungal MAMPs are chitin (Kaku et al., 2006) and ß-glucan (Cheong and Hahn, 1991). MAMP recognition is mediated by pattern recognition receptors (PRRs) located at the cell surface (Macho and Zipfel, 2014) such as the CERK1 chitin receptor of *Arabidopsis* (Miya et al., 2007). Forward genetics in *Arabidopsis* identified the receptor-like kinase MIK2 as a potential PRR and as a crucial component to recognize and respond to MAMPs from Fo (Coleman et al., 2019). PRRs are mainly expressed in root zones vulnerable to pathogen entry resulting in a heterogenic and tissue-specific responsiveness to different MAMPs (Chuberre et al., 2018). Responsiveness to chitin, for instance, is mostly confined to the mature zone and other parts of the root system are relatively insensitive to this MAMP and do not mount immune responses upon exposure to chitin (Millet et al., 2010; Coleman et al., 2019). This heterogeneity could explain why Fol typically does not penetrate mature root zones (Mes et al., 2000). In the responsive zones MAMP recognition results in activation of pattern-triggered immunity (PTI), which confers resistance to a wide variety of potential pathogens (Jones and Dangl, 2006; Bigeard et al., 2015).

**Figure 1:**
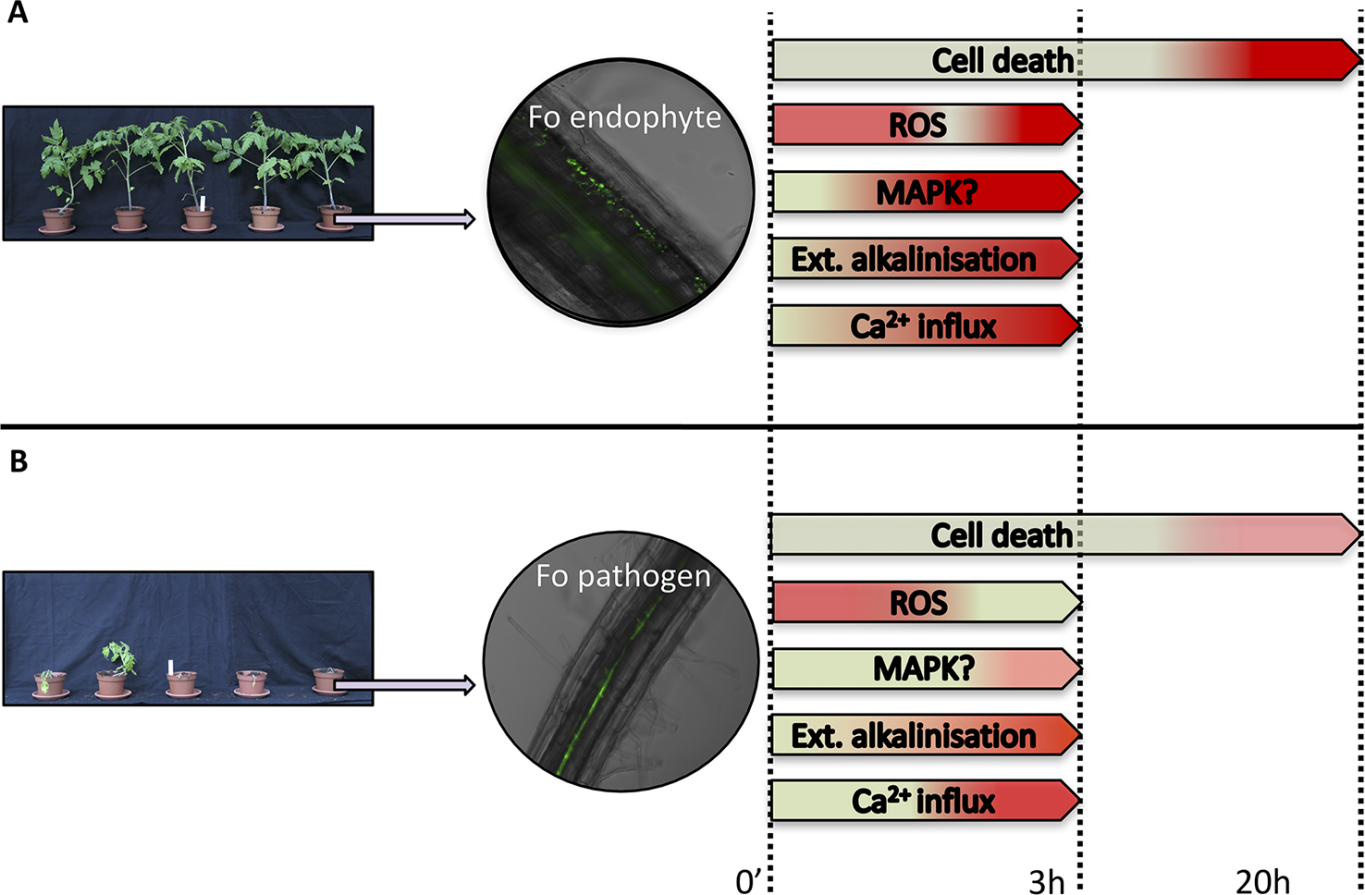
Schematic representation of plant responses upon *Fusarium oxysporum* (Fo) inoculation. Responses of plants following exposure to a Fo endophyte **(A)** or a pathogen **(B)**. Pictures on the left show representative phenotypes of tomato plants upon inoculation with either an endophytic or a pathogenic Fo strain. Middle panels show root colonization by GFP-labeled Fo strains visualised by fluorescence microscopy. The right panel summarizes early signaling responses upon Fo exposure to plant cell cultures (Olivain et al., 2003; Humbert et al., 2015). The response amplitudes are color-coded from green (lowest) to red (highest).

Activation of PTI induces a variety of early signaling responses, such as a cellular Ca^2+^ and H^+^ influx resulting in extracellular alkalinization, production of reactive oxygen species (ROS), and phosphorylation of mitogen-associated protein kinases (MAPKs) (Bigeard et al., 2015; Chuberre et al., 2018). A number of PTI-associated signaling responses that have been monitored in cell cultures upon Fo treatment are depicted in **Figure 1**. Cell cultures have been instrumental to study early plant responses to Fo (Olivain et al., 2003; Humbert et al., 2015). Flax cell cultures exposed to germinated microconidia of Fo47 show a stronger extracellular alkalinization response than cells treated with pathogenic Fo f.sp. *lini* (Foln) (**Figure 1**) (Olivain et al., 2003). Also the Ca^2+^ influx was higher upon Fo47 exposure than to Foln application (Olivain et al., 2003). Ca^2+^ influx activates calmodulin (CaM), and in cucumber roots the CaM signal transduction pathway was more strongly induced upon CS-20 colonization than when treated with pathogenic Foc (Pu et al., 2014), implying a weaker PTI induction by pathogenic strains.

ROS, besides being signaling molecules, have direct toxic effects on microbes (O'Brien et al., 2012) and can induce cell death thereby limiting progression of biotrophic pathogens (Mur et al., 2008). Within minutes Fo47 and Foln induce a similar early ROS burst in flax cells (Olivain et al., 2003). Fo47, however, also triggers a second more vigorous burst 3 h post-exposure, which is absent upon Foln treatment. Fo47 also induced more cell death than Foln especially at 14 hpi. A similar observation was made using tomato cell cultures incubated with germinated microconidia of Fo47 or Fol (Humbert et al., 2015). Analogously, inoculation of non-pathogenic Fo that triggers EMR against pathogenic Foa in asparagus induced a cell death response (≈10% cell death) in roots while no cell death was detected when Foa alone was inoculated (He et al., 2002). Transcriptome analysis of soybean roots infected by pathogenic Fo pathogen revealed upregulation of several MAPKs at a relative late stage (72 hpi) of infection, while none was induced by an endophytic strain (Lanubile et al., 2015). Whether MAPKs are differentially phosphorylated in an interaction between roots and Fo endophytes or pathogens remains a question for future study. Taken together, whereas both endophytes and pathogenic Fo strains trigger early PTI signaling, these responses are typically less pronounced in the presence of the latter, suggestive of stronger immune suppression by pathogenic strains.

An effective PTI response results in a transient, local and systemic transcriptional reprogramming of the host (Boller and Felix, 2009; Millet et al., 2010; Bigeard et al., 2015; Chuberre et al., 2018). For instance, in Fo47-inoculated pepper roots a transient expression of a PR-1 protein, a chitinase and a sesquiterpene cyclase (involved in capsidiol synthesis) was observed at 48 hpi, after which expression returned to basal levels at 120 hpi (Veloso and Díaz, 2012). In tomato roots, Fo47 and Fol did not differentially affect expression of a set of PR marker genes when monitored at 48, 72, or 96 hpi: two chitinases (*CHI9* and *CHI3*), two glucanases (*GLUB* and *GLUA*), a lypoxygenase (*LOXD*) and PR-1a (Aimé et al., 2013). However, during later stages of infection at 6 to 22 days post-inoculation (dpi), Fo47, unlike Fol, did not trigger accumulation of PR transcripts (Aimé et al., 2008). In contrast, in cucumber roots CS-20 did induce major transcriptional changes, and at 72 hpi there was a strong induction of *PR3*, *LOX1*, *PAL1*, and *NPR1* and of CaMs, *CsCam7* and *CsCam12* being the strongest induced. At the same time point pathogenic Foc induced *NPR1* and to a lesser extent *PR3* and *PAL1* expression (Pu et al., 2014). RNA-seq analysis of soybean roots inoculated with endophytic or pathogenic Fo revealed that the latter induced more, and stronger, transcriptional changes at 72 and 96 hpi (Lanubile et al., 2015). The literature is ambiguous regarding transcriptional reprogramming in plant–Fo interactions, which might originate from dissimilarities in experimental setup, sampling time, and/or plant-endophyte combination (**Table 1**). Together the data shows that transcriptional reprogramming following Fo endophyte colonization varies depending on the strain, but typically is transient and returns to basal levels within days. The observation that CS-20 affects transcriptional responses more strongly than Fo47 correlates with CS-20 being a more potent EMR-inducer (Larkin and Fravel, 1999). Xylem sap proteome analysis of susceptible tomato plants showed a significant change in abundance of up to 92% of the identified proteins at 2 weeks post-inoculation of Fol (Gawehns et al., 2015; de Lamo et al., 2018). Among the proteins showing the highest induction are the PR proteins PR-1 and PR-10. Contrarily, at the same time point no significant changes were detected in the proteome of Fo47-inoculated plants as compared to mock treatment (de Lamo et al., 2018). In summary, the transcriptional reprogramming in response to Fo endophytes is confined to the first days of the interaction, while pathogenic strains induce changes mostly during later stages when disease symptoms emerge. At these later stages, major changes are also detected in the xylem sap proteome of diseased tomato plants.

PTI is hypothesized to result in establishment of physicochemical barriers such as callose depositions at the cell walls and exudation of phytoalexins aimed at restricting microbial invasion (Millet et al., 2010). In Fo47-inoculated pea roots, host cell-wall penetration attempts by the endophyte appear constrained by callose-containing papillae depositions (Benhamou and Garand, 2001). Similar observations have been made in Fo–cucumber (Benhamou et al., 2002), Fo–flax (Olivain et al., 2003), and Fo–tomato interactions (Le Floch et al., 2009). Fo47 also induced accumulation of the phenolic compound caffeic acid in pepper roots at 48 hpi (Veloso et al., 2016), whereas in tomato roots CS-20 induced accumulation of ferulic acid at 72 hpi (Panina et al., 2007). Both compounds have *in vitro* antimicrobial activity to *Verticillium dahliae* (Veloso et al., 2016). Pea roots colonized by Fo47 respond by formation of an osmiophilic compound coating the secondary wall and the pit membranes of the vessel lumen (Benhamou and Garand, 2001). Inoculation with pathogenic Fo f.sp. *pisi* did not trigger these types of responses in pea (Benhamou and Garand, 2001).

Taken together, both Fo endophytes and pathogens trigger local PTI responses but these appear suppressed/evaded by the latter, likely by the secretion of host-specific effectors. Indeed, Fo effectors have been identified that suppress PTI, a prime example being Fol Avr2 that suppresses ROS production, callose deposition, MAPK phosphorylation, and growth-inhibition upon MAMP application (Di et al., 2017b). Recently, a chitin deacetylase (PDA1) has been found to be required for pathogenicity of Fo f.sp. *vasinfectum* to cotton (Gao et al., 2019). This provides evidence of a PTI avoidance strategy as de-acetylation of chitin converts it into chitosan, which is a poor inducer of PTI (Gao et al., 2019). Another strategy to evade PTI activation is masking fungal MAMPs. LysM-containing effectors in *Cladosporium fulvum* are involved in chitin-binding, thereby preventing their perception by the host (Bolton et al., 2008). LysM domain-containing effector genes are also present in Fo genomes (de Jonge et al., 2010; de Sain and Rep, 2015) and a LysM-containing protein secreted by Fol has been identified in tomato xylem sap (Gawehns et al., 2015; de Lamo et al., 2018). However, further research should clarify whether its role in pathogenicity is similar to that of *C. fulvum*. In summary, successful suppression of PTI by Fo pathogens seems required to cause disease and strains unable to do so, e.g. because they lack the proper host-specific effectors, do not cause disease and exert endophytic lifestyles.

## EMR Involves Localized Cell Death and Accumulation of Specific PR Proteins in the Xylem Sap

The molecular and physiological changes in roots during EMR have been studied in some detail and were mostly focused on changes in transcriptome, metabolome, and xylem sap proteome. Whereas Fo endophytes typically trigger an early, minor, and transient change in gene expression, pathogenic strains induce a major and later (days) transcriptional reprogramming during the onset and development of disease (see *The Timing and Amplitude of Root Responses Upon Colonization by Endophytic or Pathogenic F. oxysporum Differ*). In tri-partite interactions surprisingly little changes in gene expression have been reported. One study of Fo47-inoculated tomato roots challenged with Fol revealed induction of transcripts encoding an acidic extracellular chitinase (*CHI3*), an acidic extracellular ß-1,3-glucanase (*GLUA*), and *PR-1a* 48 h after inoculation (Aimé et al., 2013).

Metabolomic studies revealed that pre-treatment of pepper plants with Fo47 2 days prior to *V. dahliae* inoculation enhanced the accumulation (at 8 and 24 hpi) of a phenolic acid, chlorogenic acid, in the roots in response to the latter (Veloso et al., 2016). Phenolic acids are involved in fortification of cell walls when cross-linked to cell wall polymers by a ROS-catalyzed process (McLusky et al., 1999; Bubna et al., 2011; O'Brien et al., 2012). Phenolics, together with callose, ROS, peroxidases, and structural proteins form the major constituents of the papillae depositions that are proposed to block cell entry of Fo (Underwood, 2012). In agreement, pre-treatment of cucumber roots with Fo47 resulted in more papillae depositions preventing *P. ultimum* to penetrate host cells (Benhamou et al., 2002). Another physiological aspect of EMR is the endophyte-induced host cell death during early stages of colonization. This phenomenon seems to be common among non-pathogenic strains as Fo endophytes typically induce host cell death in the root cortex to a larger extent than Fo pathogens during early stages of infection (He et al., 2002; Olivain et al., 2003; Humbert et al., 2015; Gordon, 2017). Noteworthy, in a mutagenesis screen of different Fo endophytes, those losing their ability to trigger biocontrol also showed a reduced induction of host cell death in cell cultures despite retaining its host colonization capabilities (Trouvelot et al., 2002; L'Haridon et al., 2007; Alabouvette et al., 2009).

Pathogenic Fo strains show reduced vasculature colonization upon EMR induction, which might be caused by a change in the xylem sap proteome. To address this hypothesis the xylem sap proteome of tomato plants inoculated with Fo47 and/or Fol was compared (de Lamo et al., 2018). Of the 388 quantifiable proteins, the abundance of only two proteins was strongly increased in the tri-partite interaction as compared to the mock controls. Accumulation of these two proteins, a β-glucanase and NP24, was induced 45- and 33-fold respectively as compared to the control. β-Glucanases exert its antimicrobial activity by hydrolyzing glucan molecules, one of the most abundant polysaccharides in fungal cell walls (Stintzi et al., 1993). Furthermore, the released ß-1,6-glucans act as fungus- and oomycete-specific MAMPs triggering host immune responses (Fesel and Zuccaro, 2016). NP24 is a member of the PR-5 family that includes osmotin and thaumatin-like proteins (Stintzi et al., 1993; Liu et al., 2010). PR-5 proteins exert *in planta* antimicrobial activity against the pathogens *Phytophthora infestans* (Woloshuk et al., 1991), *P. capsici*, and Fo (Mani et al., 2012) by disrupting their plasma membrane integrity *via* the formation of pores (Vigers et al., 1992). In addition, some PR-5 proteins exert ß-1,3-glucanase activity that could contribute to their antimicrobial activity (Grenier et al., 1999; Menu-Bouaouiche et al., 2003). Besides a direct effect on the pathogen, overexpression of a plum PR-5 in *Arabidopsis* activated the production of the phytoalexin camalexin (El-kereamy et al., 2011). The correlation between EMR and NP24 abundance is intriguing, as the only differentially accumulated protein in the xylem sap of resistant tomato plants inoculated with an avirulent Fol strain is also a PR-5 family member. Accumulation of this xylem sap-specific PR-5x protein was induced 158-fold upon inoculation of the avirulent pathogen. In a compatible interaction the abundance of the protein also increased, but to a much lower extent (de Lamo et al., 2018). The finding that PR-5 isoforms also specifically accumulate in xylem sap of susceptible and resistant *Brassica oleracea* infected with Fo f.sp. *conglutinans* (Pu et al., 2016) further indicates a role for these proteins in controlling the proliferation of pathogenic Fo strains in the vasculature. The observation that pathogenicity-compromised Fol strains in which specific effectors are deleted trigger an >200-fold induction of NP24 in the xylem sap provides additional support for this hypothesis (Gawehns et al., 2015). How these Fol effectors affect accumulation of PR-5 isoforms in tomato is unknown, but various plant pathogens, including *V. dahliae* (Zhang et al., 2019), *Blumeria graminis* (Pennington et al., 2016), and *B. cinerea* (Gonzalez et al., 2017), secrete effectors that directly target PR-5 proteins, stressing their importance in plant fungal interactions.

Altogether, Fo-based EMR seems to be a root-mediated response that triggers, among other responses, specific accumulation of xylem sap-localized PR-5 and ß-glucanase proteins and secretion of phenolic compounds that together with ROS are involved in cell wall lignification and callose depositions. Furthermore, host cell death induced by Fo endophytes correlates with the induction of an effective EMR response.

## EMR is Distinct From Induced Systemic Resistance and Systemic Acquired Resistance Responses

Many studies attribute Fo-induced resistance response in plants as the main contributor to biocontrol (**Table 1**). Split-root systems, in which the Fo endophyte is spatially separated from the pathogen, have shown that EMR can act systemically in root tissues (Kroon et al., 1991; Fuchs et al., 1997; Duijff et al., 1998; Larkin and Fravel, 1999; Kaur and Singh, 2007; Pantelides et al., 2009; Aïcha et al., 2014). The mechanism that transduce this signal to distant root tissues is unknown. Root colonization by endophytes such as *S. indica* or *Trichoderma* spp. triggers an induced systemic resistance response (ISR) that relies on the phytohormones jasmonic acid (JA) and ethylene (ET) (Shoresh et al., 2010; Franken, 2012). Other microbes, especially avirulent pathogens, can trigger a salicylic acid (SA)-dependent immune response, which results in systemic acquired resistance (SAR) (Pieterse et al., 2014). Both systemic responses prime the plant to respond faster and stronger to subsequent pathogen attack, thereby reducing it susceptibility to foliage-attacking pathogens (Durrant and Dong, 2004; Fu and Dong, 2013; Pieterse et al., 2014). The observation that Fo endophytes typically do not confer protection to pathogens attacking above-ground tissues (**Table 1**) raises the question whether EMR mechanistically differs from ISR or SAR. Only two reports describe Fo-induced resistance to a foliar pathogens (Biles and Martyn, 1989; Díaz et al., 2005). It was reported that a Fol strain that is pathogenic on tomato reduced susceptibility to *B. cinerea* in pepper (Díaz et al., 2005). Pre-treatment with 1-methylcyclopropene, an inhibitor of ET perception, compromised this Fol-induced plant protection to the fungus. The involvement of ET in this response implies that the non-host pathogen Fol can trigger ISR in pepper. Remarkably, Fo47 inoculation did not confer protection against *B. cinerea* in the same experimental setup, although this strain triggered EMR (Veloso and Díaz, 2012), suggesting that Fol triggers both. The other example details the cucumber-pathogen Foc that induced systemic responses in aerial tissues in watermelon (Biles and Martyn, 1989). It will be interesting to investigate whether both pathogenic Fo strains carry effectors that are recognized by these non-host plants responsible for triggering a systemic ISR-type immune response. These examples imply that non-host pathogenic Fo strains can induce both ISR and EMR, while purely endophytic strains trigger only the latter response.

Whereas tomato mutants compromised in SA signaling are hypersensitive to *Fusarium* wilt disease, an increased tolerance was observed in mutants affected in ET biosynthesis or perception (Di et al., 2017a). In contrast, susceptibility of tomato mutants deficient in JA biosynthesis towards Fol was unaffected, showing that these three phytohormones have distinct roles in the interaction between tomato and pathogenic Fo (Di et al., 2017a). The interaction between these phytohormones and Fo is complex and differs for different pathosystems (Di et al., 2016). To elucidate the role of these defense phytohormones in EMR, Constantin and co-workers analyzed Fo47-induced immune responses in wild-type tomato plants and in mutants compromised in ET, JA or SA signaling (Constantin et al., 2019). Expression of ET marker genes (*Pti4* and *ETR4*) was not induced in a tri-partite tomato–Fol–Fo47 interaction suggesting that ET is not involved in EMR (Constantin et al., 2019). Indeed, EMR was intact in tomato lines affected in either their ability to sense- (*never-ripe* mutant) or produce ET (transgenic lines constitutive expressing *ACC deaminase*) (Constantin et al., 2019). Also tomato plants with a defect in JA biosynthesis (*def1*) were still capable of mounting EMR upon co-inoculation with Fo47 and Fol. These findings make involvement of ISR in EMR unlikely, as this response requires intact ET/JA signaling pathways (Pieterse et al., 2014). Likewise, SAR, which requires SA, appears not to be involved as tomato lines compromised in SA accumulation (expressing *NahG*) exert a functional EMR response against Fol (Constantin et al., 2019). Together, these findings support a model in which EMR induced by Fo47 is distinct from ISR and SAR, as these responses require either JA/ET or SA and result in induced resistance in shoots, unlike EMR that is mostly root confined.

## Discussion

**Figure 2:**
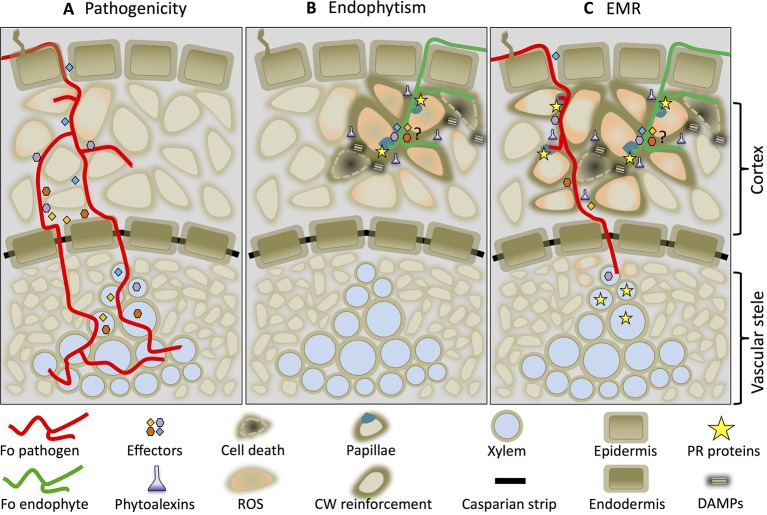
Endophyte-mediated resistance (EMR) working model. Cross-section of a root colonized by a Fo pathogen (red line) **(A)**, an endophyte (green line) **(B)**, or by both in a tri-partite interaction in which EMR is triggered **(C)**. The drawings depict an interaction around 2 days after inoculation.

Based on the data presented we propose a mechanistic model on how Fo-induced EMR prevents disease. **Figure 2** illustrates an early (≈ two dpi) interaction between a root and Fo. Both pathogenic and non-pathogenic Fo strains colonize the root epidermis and cortex. Whereas pathogenic Fo strains effectively compromise immune signaling by secreting effector proteins (**Figure 2A**) endophytes are unable to do so and trigger immune activation (**Figure 2B**). The transient induction of immune signaling confines the non-pathogenic fungus to the root cortex and restricts its growth by preventing entry into host cells by the formation of papillae and cell wall fortifications. Together these responses prevent the fungus from reaching the vasculature and causing disease. Localized cell death induced by Fo endophytes (He et al., 2002; Alabouvette et al., 2009) appears to be involved in the induction of EMR, because Fo mutants that lost their ability to induce cell death are also unable to trigger EMR even though they can still colonize the roots (Alabouvette et al., 2009). Endophytes such as *Harpophora oryzae* or the phylogenetically distant basidiomycete *S. indica*, are also known to trigger localized cell death upon root colonization (Deshmukh et al., 2006; Su et al., 2013). It is tempting to speculate that induction of host cell death by endophytes may be a generic property required for EMR induction. Possibly cell death primes, or potentiates, immune responses to an extent that they can no longer be mitigated by the effectors secreted by the pathogen. The potentiated immune responses restrict pathogen development in a tri-partite interactions and results in a reduced xylem sap colonization (**Figure 2C**). Although pathogenic Fo strains colonize the vasculature in tri-partite interactions their proliferation is reduced as are the disease symptoms. We speculate that the reduced ability to colonize the vasculature is in part due to the increased abundance of PR-5 protein family members and plant-produced ß-glucanases. Assessing the biocontrol properties of Fo in plants in which these genes are knocked-out can put this hypothesis to the test.

## Conclusion

Although being studied for over more than three decades the mechanism underlying EMR remains elusive. Understanding this inducible defense mechanism, which confers protection against root-invading vascular pathogens, holds potential for improved control of wilt diseases without affecting the conventional defense pathways. Future studies focusing on the nature of the systemic signal, the role of secondary metabolites, PR protein production, and papillae formation in tri-partite interactions will be instrumental to get a better understanding of the mechanism underlying EMR. Elucidating the relation between localized host cell death and EMR will reveal whether damage merely amplifies, or is essential, to trigger this immune response. Studying the potential role of host-specific and generic effector candidates in modulating EMR will increase our understanding of the endophytic side of the interaction, possibly allowing selection of endophytic strains conferring robust biocontrol in agricultural settings. A concern is that horizontal chromosome transfer from pathogenic Fo strains to the applied Fo endophytes could turn the latter into pathogens. Whether chromosome transfer occurs in natural setting should be investigated before agricultural application.

## Author Contributions

FL and FT conceived and wrote the manuscript.

## Funding

FL and FT are supported by the BestPass project. This project is funded by the European Union’s Horizon 2020 research and innovation program under the Marie Skłodowska-Curie grant agreement No. 676480 (International Training Network BestPass). FT also is supported by the NWO-Earth and Life Sciences funded VICI project No. 865.14.003.

## Conflict of Interest

The authors declare that the research was conducted in the absence of any commercial or financial relationships that could be construed as a potential conflict of interest.
